# Cold hardiness and biochemical response to low temperature of the unfed bush tick *Haemaphysalis longicornis* (Acari: Ixodidae)

**DOI:** 10.1186/1756-3305-7-346

**Published:** 2014-07-26

**Authors:** Zhi-Jun Yu, Yu-Lan Lu, Xiao-Long Yang, Jie Chen, Hui Wang, Duo Wang, Jing-Ze Liu

**Affiliations:** Key Laboratory of Animal Physiology, Biochemistry and Molecular Biology of Hebei province, College of Life Sciences, Hebei Normal University, Shijiazhuang, Hebei 050016 China; Department of Biology, Langfang Normal College, Langfang, Hebei 065000 China

**Keywords:** Super-cooling capacity, Cold hardiness, Acclimation, *Haemaphysalis longicornis*

## Abstract

**Background:**

The survival of overwintering ticks, is critical for their subsequent population dynamics in the spring, and consequent transmission of tick-borne diseases. Survival is largely influenced by the severity of the winter temperatures and their degree of cold hardiness at the overwintering stage. The bush tick *Haemaphysalis longicornis*, is widely distributed in China, and can transmit various pathogens that pose serious medical/veterinary problems. In the present study we investigated the effect of low temperature stress to tick survival, super-cooling point and body content of water, glycerol and total protein.

**Methods:**

After various temperature acclimations, the super-cooling point was measured by Ni/CrNi-thermocouples with a precision temperature recorder. Water content was determined from weight loss of the sample exposed to 60°C for 48 h. Glycerol content was determined using Free Glycerol Reagent as directed by the manufacturer, and total protein was determined using the Bradford assay.

**Results:**

The 50% mortality temperatures for the adults and nymphs were -13.7°C and -15.2°C, respectively; and the discriminating temperatures for the adults and nymphs were -16.0°C and -17.0°C, respectively. The super-cooling points of the adults and nymphs were -19.0°C and -22.7°C, respectively. The water content of adult *H. longicornis* decreased substantially after acclimation at 0°C for 10 d, whereas the nymphs decreased after acclimation at 0°C for 20 d, and the glycerol and proteins of both nymphs and adults were significantly increased (*p* < 0.01) when stressed at 0°C for 10 d.

**Conclusions:**

In *H. longicornis*, low temperature stress can enhance its cold hardiness and trigger appropriate responses, including reducing water content, and increasing glycerol and total protein content.

## Background

Many overwintering arthropods, including mites and ticks, are threatened with death by the low temperatures that occur during mid-winter in temperate and cold regions [1, 2]. Most of them employ various techniques to improve their cold hardiness under low-temperature conditions [3, 4]. Some enter diapause, which may or may not increase cold hardiness [5]. Ticks, as obligate blood sucking ectoparasites of terrestrial vertebrates, are widely distributed on every continent. They transmit a greater variety of pathogens (viruses, bacteria, rickettsiae, helminths and protozoa) than even mosquitoes [6]. However, the development and survival of ticks are largely dependent on the complex combination of environmental temperature and other climate variables [7]. The survival of overwintering ticks is obviously critical for their subsequent population dynamics in the spring [8]. Before the arrival of winter, ticks adopt behaviors and physiological adjustments to promote overwintering. These include searching for suitable habitation sites under leaf litter or stones [9], entering diapause, and increasing the concentration of cryprotectants like sorbitol, glycerol and various antifreeze proteins [10]. Previous investigations focused on the super-cooling capacity and cold hardiness of ticks [11–14] demonstrating that most ticks if not all, were as freeze-intolerant as many other arachnids. However, they did show a high potential super-cooling, irrespective of their geographical origin [12]. The bush tick *Haemaphysalis longicornis*, is the major vector of *Theileria spp*., *Coxiella burnetti* [15, 16], *Babesia sp*. [17], *Anaplasma phagocytophilum* [18] and a suspected vector of the notorious bunyavirus, which has caused many deaths in China [19], Japan and Korea recently [20]. *H. longicornis* is endemic to north China [21], Australia, New Zealand, Korea and Japan [22, 23], and can cause severe damage to human health and livestock production. In the field, *H. longicornis* completes one generation per year with some population overlap between developmental stages. Most survive the cold winter as nymphs under the leaf litter, with a small overwintering population of adults surviving on the host without feeding or under the leaf litter [24]. However, little is known about the physiological responses, and biochemical changes in unfed *H. longicornis* when exposed to low temperatures in winter.

In this study, *H. longicornis* individuals were cold acclimated to a series of low temperatures, and the survival rate, super-cooling capacity, and changes in water content, glycerol content and total protein were determined.

## Methods

### Collection and rearing of ticks

All ticks used in this study originated from adult *H. longicornis* collected from vegetation by blanket dragging in Xiaowutai National Nature Reserve Area (39°50′ to 40°07′N, 114°47′ to 115°30′E) of Hebei province, north China. Colonies of these ticks were fed on rabbits as described by Liu *et al*. [25], and maintained in our laboratory incubator (26 ± 1°C, 85 ± 5%RH, 6:18 (L:D)). Wild caught ticks were reared for two generations in the laboratory, with unfed nymphs (2 weeks after moulting) and adults (8 weeks following moulting) that within normal body-weight ranges randomly selected for the assays described in this study.

### Low temperature survival of unfed nymphs and adults

To determine the temperature resulting in 50% mortality (LT50) and the discriminating temperature (the temperature resulting in ~15-20% survival), which is important for testing the response of the rapid cold acclimation [26] of the unfed nymphal and adult *H. longicornis*, the survival of the ticks following a brief period of acclimation (2 h) was recorded and calculated as follows. Unfed nymphs or adults were randomly selected and placed in separate plastic vials (each vial contained 20 ticks, and each experiment was replicated 3 times) and used for each temperature treatment. The vials were transferred from the colony incubator (26 ± 1°C, 85 ± 5%RH, 6:18 (L:D)) to a series of low temperature conditions (ranging from -6°C ~ -22°C, 1°C intervals, 6:18 (L:D)). After 2 h exposure to each low temperature, each group of ticks was immediately returned to the colony incubator. The percent survival at each temperature was recorded after 24 h recovery in the colony incubator, and the ticks that were able to coordinate their movements were recognized as survivors. The survival rate of ticks that were not exposed to low temperature (i.e. those kept in the colony incubator) served as the control.

### Effects of short term acclimation of nymphs to various temperatures

To determine the effects of rapid cold acclimation, unfed nymphs (20 per group) were confined to plastic vials covered with gauze, and transferred from the colony incubator directly to an incubator set to -3°C, 0°C, 5°C, 10°C, 15°C or 20°C (85 ± 5% RH, 6 L:18D). Ticks were held at the selected temperature for 1 h, 2 h, 3 h or 4 h, respectively, and then transferred to their discriminating temperature, which was determined in the section just above, and chilled for 2 h. After this, all ticks were returned to the colony incubator (26 ± 1°C, 85 ± 5%RH, 6:18 (L:D)) and held for 24 h. Survival rate was then assessed as described above. Controls were ticks transferred directly from the colony incubator to their discriminating temperature.

### Cold hardiness of adult and nymphal *H. longicornis*

The cold hardiness of nymphal and adult *H. longicornis* was evaluated by exposing the ticks to a range of temperatures for 48 h and recording their survival. Each sample consisted of 50 nymphs or adults (without sex determination) kept in a glass tube with a cotton plug. Groups of ticks were put into a freezer, or incubator, and exposed to a constant temperature. All temperatures between -20°C and +20°C were tested, at 5°C intervals. Temperature fluctuations inside the test tubes did not exceed ±0.5°C, as measured by Ni/CrNi-thermocouples with a precision temperature recorder (Jiangsu Senyi Developmental Company, China). After 48 h of exposure, the ticks were returned to the colony incubator for 24 h, and then examined to determine survival. The experiment was conducted in triplicate.

### Determination of the super-cooling point for unfed nymphs and adults

Short and long term acclimation was achieved as follows. For short term acclimation, randomly selected unfed nymphal and adult *H. longicornis* were transferred from the colony incubator and exposed to fixed temperatures of 0°C or 5°C for 2 h to cold shock the ticks. For the long term acclimation, both unfed nymphal and adult ticks were exposed to 0°C for 10 d or 20 d. The super-cooling point of the nymphal and adults ticks was determined by attaching Ni/CrNi-thermocouples to their dorsal surface using paraffin wax. The tick-thermocouple arrangements were fixed inside polyethylene tubes, which were then placed into an aluminum cooling block. The cooling block was then transferred to a refrigerated circulating bath (Thermo Scientific NESLAB RTE-740, USA). The temperature was decreased at a rate of 0.5°C/min, from an initial temperature of 26°C. The body temperatures of the ticks were recorded at 1 s intervals using a precision temperature recorder (Jiangsu Senyi Developmental Company, China). The super-cooling point was defined as the lowest body temperature reached prior to the formation of ice crystals in the body. This point could be seen as a small peak on a scatter plot graph of the recoded data, which indicates the heat released during the phase change and means of the emission of the exotherm [27]. A minimum of 30 ticks were used in each group for super-cooling point determination.

### Changes in water content, glycerol and total protein of ticks after short and long term acclimation

To induce short and long term acclimation, groups of unfed ticks (100 nymphs or 50 adults) were selected such that the initial mean weight did not differ statistically between the groups. They were exposed to 0°C for 2 h, 5°C for 2 h, 0°C for 10 d or 0°C for 20 d before determining the content of water, glycerol and total protein.

After short and long term acclimation, the ticks were tested for survival, weighed, dried for 48 h at 60°C, and their dry weights were recorded. Preliminary experiments determined that no further weight loss occurred beyond 48 h. The water content was calculated for each tick based on its weight loss. To determine the amount of glycerol and proteins, adult and nymphal frozen ticks were crushed and homogenized in 1 mL phosphate buffered saline (PBS, 0.01 mol/L, pH 7.4), and the homogenates were centrifuged at 10, 000 rpm for 15 min in 4°C. The pelleted fraction was cleaned using 0.5 mL PBS, re-centrifuged, and the supernatant combined with that from the previous centrifugation. Glycerol concentrations were assayed using Free Glycerol Reagent (Sigma-Aldrich) according to the manufacturer’s protocol. Protein content was determined by Bradford assay [28] with absorbance measured at 595 nm.

### Statistical analysis

Statistical analysis was performed using STATISTICA Version 6.0 (StatSoft, Inc., Tulsa, OK, U.S.A.). All parametric data comparisons were performed by one-way analysis of variance (ANOVA) and probit analysis was used to calculate the LT50.

### Ethical approval

All the experiments were approved by the Animal Ethics Committee of Hebei Normal University.

## Results

### Survival of ticks held at low temperatures

**Figure 1:**
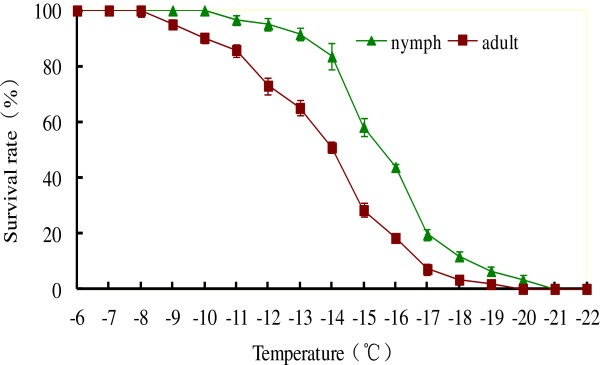
**Survival rate of nymphal and adult** ***H*** **.** ***longicornis*** **after being cold shocked for 2 h at a series of low temperatures from -6°C to -22°C.**

After cold shocking ticks for 2 h over a range of subzero temperatures, the survival rate gradually declined, corresponding to the decrease in temperature, beginning at -8°C and -10°C for adults and nymphs respectively (Figure 1). None of the adults survived at -20°C, and all the nymphs died at -21°C. Nymphs held at -13°C, -15°C or -17°C had survival rates of 91.5%, 58.2%, and 19.6%, respectively. Survival rate of the adults at -11°C, -14°C and -16°C was 85.8%, 50.8% and 18.3%, respectively (Figure 1). The 50% mortality temperature calculated for the adults and nymphs was -13.7°C and -15.2°C, respectively. The discriminating temperature for the adults and nymphs was -16.0°C and -17.0°C, respectively.

### Effects of short term acclimation of nymphs to cold temperatures

**Figure 2:**
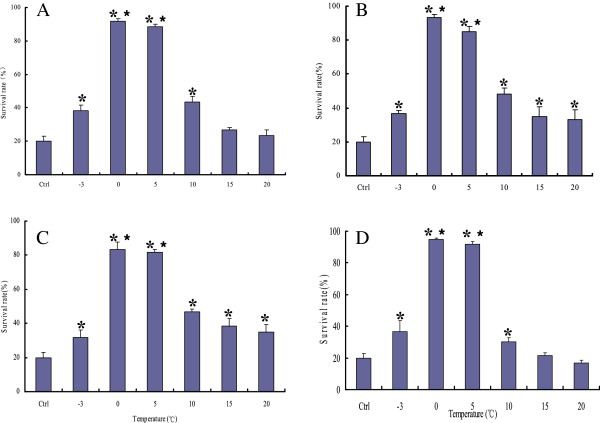
**Survival rate of nymphal** ***H*** **.** ***longicornis*** **after being cold acclimated for a certain time and stressed at the discriminating temperature. A**: acclimate for 1 h; **B**: acclimate for 2 h; **C**: acclimate for 3 h; **D**: acclimate for 4 h. The different superscripts indicates statistical difference, *p < 0.05, **p < 0.01. Ctrl: Control group.

Short term acclimation in nymphs was induced by acclimating at temperatures ranging from -3°C to 20°C for a certain time (1–4 h), and then stressed at the discriminating temperature for 2 h. The subsequent survival rate was significantly increased following cold acclimation at -3°C (*p* < 0.05), 0°C (*p* < 0.01), 5°C (*p* < 0.01) and 10°C (*p* < 0.05) for a period of 1 h, 2 h, 3 h or 4 h, compared to the non-acclimated control group (20.3 ± 2.9%) (Figure 2). Maximal enhancement of cold tolerance was induced by acclimation to 0°C and 5°C, while acclimation at 15°C (*p* < 0.05) and 20°C (*p* < 0.05) for 3 h or 4 h could also enhance their cold tolerance (Figure 2).

### Cold hardiness of *H. longicornis*

**Figure 3:**
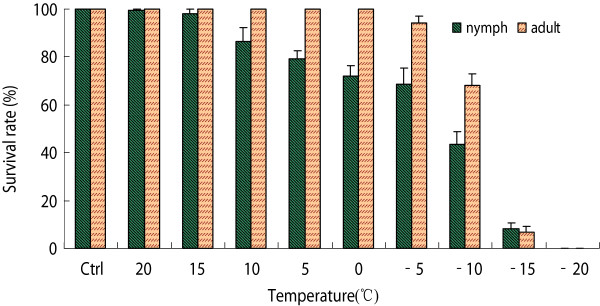
**The survival rate of** ***H*** **.** ***longicornis*** **after direct exposure to a series of low temperatures for 48 h.**

The cold hardiness of *H. longicornis* was evaluated by calculating the survival rate of the ticks treated at a series of temperatures for 48 h. When exposed to temperatures at or above 0°C, 100% of the acclimated adult ticks survived, compared to 73% of nymphs. Nymphal mortality increased gradually as temperatures decreased from 15°C to -10°C, whereas adult survival began to decrease at temperatures less than -5°C (Figure 3). The survival of nymphs was higher than that of adults when held at -15°C (*p* < 0.05). No ticks survived after being held at -20°C.

### Super-cooling capacity of *H. longicornis*

The super-cooling points of the nymphs and adults were -22.7 ± 1.4°C and -19.0 ± 3.7°C, respectively. In short term acclimation, the super-cooling point of nymphs that had been cold acclimated at 0°C (*p* < 0.05) or 5°C (*p* < 0.05) for 2 h, was significantly lower than that of the control group (Table 1). There were no significant differences between the groups acclimated at 0°C or 5°C for 2 h (*p* > 0.05). For the long-term acclimation, nymphs that had been cold acclimated at 0°C for 20 d had a significantly lower super-cooling point compared to the control group (*p* < 0.05). However, there was no difference between the control group and those nymphs acclimated for only 10 d (*p* > 0.05; Table 1).

**Table 1:** **The physiological and biochemical responses of nymphal** ***H*** **.** ***longicornis*** **after low-temperature treatments**

Treatment	Physiological and biochemical response (nymph)				
	Survival rate (%)	Super-cooling point (°C)	Water (%)	Glycerol (μg/mL)	Protein (μg/mL)
Control	100 ± 0.0	-22.7 ± 1.4	57.6 ± 1.0	2.6 ± 0.1	68.2 ± 7.9
0°C 2 h	100 ± 0.0	-23.8 ± 0.9^*^	59.3 ± 1.6	2.9 ± 1.1	53.9 ± 12.7
5°C 2 h	100 ± 0.0	-24.0 ± 1.4^*^	64.8 ± 1.1^**^	2.9 ± 0.3	61.0 ± 8.6
0°C 10d	62.4 ± 5.6	-23.3 ± 1.4	54.7 ± 2.2	4.2 ± 0.1^**^	112.5 ± 4.6^**^
0°C 20d	54.3 ± 7.8	-23.7 ± 1.1^*^	50.3 ± 1.6^**^	3.5 ± 0.3^**^	21.8 ± 6.6^**^

^*^ *p* < 0.05, ^**^ *p* < 0.01.

When compared to the controls, the super-cooling point of the adults acclimated at 0°C (*p* < 0.01) or 5°C (*p* < 0.01) for 2 h was significantly lower. For the long-term acclimation, the super-cooling point of the adults acclimated at 0°C for 10 d or 20 d was significantly lower compared to the non-acclimated controls (Table 2).

**Table 2:** **The physiological and biochemical responses of adult** ***H*** **.** ***longicornis*** **after low-temperature treatments**

Treatment	Physiological and biochemical response (adult)				
	Survival rate (%)	Super-cooling point (°C)	Water (%)	Glycerol (μg/mL)	Protein (μg/mL)
Control	100 ± 0.0	-19.0 ± 3.7	56.7 ± 0.6	1.1 ± 0.1	29.8 ± 2.3
0°C 2 h	100 ± 0.0	-19.8 ± 2. 7^**^	63.3 ± 1.4^**^	1.0 ± 0.1	11.8 ± 1.7^*^
5°C 2 h	100 ± 0.0	-20.3 ± 2.4^**^	62.2 ± 2.1^**^	1.1 ± 0.2	10.8 ± 4.8^*^
0°C 10d	98.2 ± 0.7	-20.7 ± 2.3^**^	49.6 ± 3.4^**^	3.1 ± 0.4^**^	59.3 ± 9.8^**^
0°C 20d	96.5 ± 1.2	-21.3 ± 1.8^**^	48.4 ± 1.0^**^	4.4 ± 0.6^**^	20.0 ± 4.8

^*^ *p* < 0.05, ^**^ *p* < 0.01.

### Changes in water content, glycerol and total protein of nymphal and adult *H. longicornis*

Changes in water content, glycerol and total protein of nymphal *H. longicornis* were determined after a series of cold temperature treatments, and results indicated that the water content of nymphs increased significantly after acclimation at 5°C for 2 h (*p* < 0.01), and significantly decreased in nymphs held at 0°C for 20 d (*p* < 0.01). There were no significant changes in nymphs acclimated at 0°C for 2 h (*p* > 0.05) or at 0°C for 10 d (*p* > 0.05) (Table 1). The glycerol content of nymphs that were acclimated at 0°C for 10 d or 20 d was significantly increased compared to both the non-acclimated control group and the other low temperature treatments (*p* < 0.01; Table 1), and no significant changes were observed when treated at 0°C or 5°C for 2 h. The protein content of nymphs significantly increased following acclimation at 0°C for 10 d when compared with that of the control group (*p* < 0.01), but decreased significantly when acclimated at 0°C for 20 d (*p* < 0.01). There was no increase in the protein content of nymphs acclimated at 0°C or 5°C for 2 h (*p* > 0.05) (Table 1).

As for the adults, water content increased significantly in the nymphs following acclimation at 0°C (*p* < 0.01) or 5°C for 2 h (*p* < 0.01), but decreased significantly in those ticks acclimated at 0°C for 10 d (*p* < 0.01) or 20 d (*p* < 0.01) (Table 2). The glycerol content increased significantly after acclimation at 0°C for 10 d or for 20 d compared to both the non-acclimated control group and the other low temperature treatments (*p* < 0.01; Table 2), and no significant changes were detected from the adults held at 0°C or 5°C for 2 h when compared to controls (*p* > 0.05) (Table 2). Adults acclimated at 0°C for 10 d (*p* < 0.01) contained significantly more protein than controls, but those adults acclimated at 0°C and 5°C for 2 h exhibited decreased protein content (*p* < 0.05). Acclimation at 0°C for 20 d had no effect on the protein content of adults (*p* > 0.05) (Table 2).

## Discussion

We demonstrate here that nymphal and adult *H. longicornis* are freeze-susceptible [11, 12, 14, 29, 30]. Nymphal *H. longicornis* are slightly more cold-hardy than the adults, the LT50 being -15.2°C and -13.7°C, respectively (Figure 1). Similarly, the discriminating temperature for the adults and nymphs was -16.0°C and -17.0°C, respectively, all indicating that nymphs have a slightly broader range of tolerance. A possible explanation could be related to the smaller body size of the nymphs, and this was consistent with our previous work that nymphal and adult *H. longicornis* are able to survive overwinter in the field [24].

The super-cooling point of the nymphs (-22.7°C) was lower than that of the adults (-19.0°C; Tables 1 and 2). A similar situation pertains to several other tick species [14, 29–32]. Although the super-cooling point seems to have no predictive value for any tick species in an ecological context, it does represent the lower temperature limit for survival [14]. In the current work, both short- and long-term acclimation decreased the super-cooling point, indicating that cold acclimation in the months prior to the onset of winter may be adaptive for winter survival in the field.

Low temperature acclimation lowers the super-cooling point in many other arthropods like *Pieris brassicae* [33] and *Monochamus alternatus* [34], and within a limited range, the lower the temperature, the greater the resulting cold hardiness. In the current study, after cold acclimated at 0°C or 5°C for 2 h, the super-cooling point of the nymphal and adult *H. longicornis* was decreased compared with the control group.

Although tick survival increased after short term acclimation, the changes in water, glycerol and total protein content were observed only after long-term cold acclimation (Tables 1 and 2). The cold tolerance of many arthropods results from an increase in various solutes [35–37]. However, some insects increase their freeze tolerance by losing water, whereas the opposite occurs in *H. longicornis* (Tables 1 and 2) and in *D. variabilis* and *A. americanum* ticks [8]. This may be attributed to quick water absorption just after the onset of low temperatures, which will hamper the desiccation in the subsequent long winter [38], and the observed water loss in the long time exposure of nymphal and adult *H. longicornis* in the current study may support this hypothesis. Additionally, glycerol and other low molecular weight substances may also protect against both desiccation and cold temperatures [39]. Although the water content of both nymphal and adult *H. longicornis* substantially decreased (*p* < 0.01) after acclimation at 0°C for 10 d or 20 d, the increased levels of glycerol and protein content may act to increase the cold hardiness and reduce water loss in the overwintering stage.

Numerous proteins, including ice-nucleating agents and the antifreeze/thermal hysteresis proteins, enhance the cold hardiness of many arthropods [40]. However, not much is known about the cryoprotectants in ticks, although many stress response proteins have been identified from transcriptomics and proteomics data [41]. In the current study, short term acclimation had no effect on the protein content in nymphs, whereas in adults, the protein content declined slightly following rapid cold treatment. However, the protein content in both nymphal and adult *H. longicornis* increased significantly after acclimation at 0°C for 10 d (Tables 1 and 2), suggesting that cryoprotective proteins might be produced by this treatment. The main objective of this study was to extend our knowledge on the complexity of the physiological adjustments linked to the cold hardiness in *H. longicornis*. Further investigations are required to confirm and characterize the proteins produced during the acclimation phase.

## Conclusions

The tick *H. longicornis* is freeze susceptible, and low temperature stress can enhance its cold hardiness and trigger a reduction in water content and increase in glycerol and proteins, suggesting that these serve as cryoprotectants.
